# The Effect of Prickly Pear, Pumpkin, and Linseed Oils on Biological Mediators of Acute Inflammation and Oxidative Stress Markers

**DOI:** 10.1155/2020/5643465

**Published:** 2020-08-02

**Authors:** Sana Bardaa, Mouna Turki, Sameh Ben Khedir, Massara Mzid, Tarek Rebai, Fatma Ayadi, Zouheir Sahnoun

**Affiliations:** ^1^Laboratory of Pharmacology, Faculty of Medicine of Sfax, University of Sfax, Tunisia; ^2^Laboratory of Biochemistry, CHU Habib Bourguiba, University of Sfax, Tunisia; ^3^Laboratory of Histology Embryology and Reproductive Biology, Faculty of Medicine of Sfax, University of Sfax, Tunisia

## Abstract

Medicinal plants have been used as a source of effective and safe alternative therapeutic agents for various ailments including inflammation. In fact, the aim of this study is to assess the topical anti-inflammatory and antioxidative potential effects of *Cucurbita pepo* (pumpkin), *Linum usitatissimum* (linseed), and *Opuntia ficus indica* (prickly pear) oils on acute inflammation using carrageenan-induced paw edema model. The study was conducted on 36 rats splitted in 6 groups: a normal control group and 5 carrageenan-treated groups (1%), each treated with either a normal saline, the reference drug (“Inflocine®” 2 mg/paw), pumpkin, linseed, or prickly pear oils (25 *μ*l/paw). The response to these treatments was mainly assessed by the measuring of edema paw size, hematological and biochemical analysis, oxidative stress testing, and histological study. All the studied seed oils especially prickly pear oil proved to be efficient in treating acute inflammation. The oil-treated groups revealed a significant (*p* < 0.05) decrease in the clinical signs of inflammation, hematological parameters (white blood cells and platelets), concentrations of CRP and fibrinogen, and congestion compared to the normal saline-treated group. The results also showed that the tested oils, endowed with a radical scavenging ability, could significantly increase the activities of SOD, CAT, and GPx in carrageenan-treated skin by reducing the lipid peroxidation and protein oxidation (TBARS, AOPP). The anti-inflammatory effect of the tested oils was closely related to both their antioxidant properties as well as their bioactive compounds (polyunsaturated fatty acids, vitamin E, and phytosterols). For the first time, the findings of the current study highlight the “*in vivo*” anti-inflammatory property of pumpkin, linseed, and prickly pear oils on carrageenan-induced acute inflammation by regulating inflammatory mediators and oxidative stress markers.

## 1. Introduction

The worldwide rise of inflammatory diseases has made them to become a global emergency. The inflammation which underlies almost every disease process has been shown to cause many common diseases. Indeed, inflammation is a protective reaction of the body to harmful stimuli [1]. Persistent and uncontrolled inflammation leads to the development of several chronic inflammatory diseases such as psoriasis, rheumatoid arthritis, and atherosclerosis [2]. Thus, it behooves researchers to develop effective anti-inflammatory treatments to control acute inflammation using medicinal plants that have been served as a source of effective and safe alternative therapeutic agents for various ailments including inflammation [3]. Accordingly, the *Cucurbita pepo* (pumpkin), *Linum usitatissimum* (linseed), and *Opuntia ficus indica* (prickly pear), that have prominent extracted seed oils, are studied as a possible anti-inflammatory treatment. The inflammatory process induces oxidative stress and reduces cellular antioxidant capacity [4]. Thus, the current study aimed at assessing and reporting the “*in vivo*” anti-inflammatory and antioxidative activities of pumpkin, linseed, and prickly pear extracted oils on acute inflammation induced by carrageenan.

Although antioxidant and antibacterial properties of pumpkin, linseed, and prickly pear oils were reported in several studies [5–7], the acute anti-inflammatory activities using carrageenan test are still not scientifically confirmed.

*Cucurbita pepo* (pumpkin) is an annual climber plant globally well-known [8]. According to several reports, pumpkin seeds contain considerable amounts of polyunsaturated fatty acids (linoleic acid), monounsaturated acid (oleic acid), phytosterols, and *α*-tocopherol with therapeutic values [5].

The therapeutic use of pumpkin was described in many traditional medicines. Previous studies showed that pumpkin seeds provided various pharmacological properties. It has been used as a remedy for diabetes and several urological disorders (benign prostatic hyperplasia and micturition problems) [8–10]. It was reported that pumpkin seed oil had interesting antioxidant, antibacterial, and wound healing activities [5, 11].

*Linum usitatissimum*, commonly known as linseed or flaxseed, is an annual plant that belongs to the family of Linaceae. Traditionally, flaxseed is grown for its oil. The linseed and its derivative are rich sources of the essential fatty acid (alpha-linolenic acid) and an array of antioxidants [6, 12]. Linseed extracted oil is used in traditional medicine as an antioxidant, laxative, sedative, and emollient agent [11, 13].

*Opuntia ficus indica* (L.) Mill, known as prickly pear, is a succulent plant that belongs to the cactaceae family. This plant is widespread in dry regions, mainly in the Mediterranean area. Prickly pear seed oil is particularly characterized by a high content of vitamin E, sterols, and fatty acids. The linoleic acid is a major polyunsaturated fatty acid, oleic acid is the dominant monounsaturated fatty acid, and palmitic acid is the major saturated fatty acid [7, 14].

It was described that the prickly pear fruit had interesting biological properties, and thus, it was widely used in traditional medicine for its antidiabetic and hypolipidemic properties [15]. Some authors attributed its possible antioxidant, antibacterial, and wound healing effects to its interesting composition [7, 16].

## 2. Material and Methods

### 2.1. Material

The *Opuntia ficus indica* L. plant seeds were harvested (November 2014) in Sbeitla (region of Kasserine, North-central Tunisia). However, the *Cucurbita pepo* L. and brown *Linum usitatissimum* seeds were harvested in November 2014 in Sidi Bouzid and Mehdia regions, respectively.

The seeds were authenticated by Dr. Hamadi Ben Salah, and the voucher sample was deposited at INRAT. The seeds had been dried according to Omega Tunisie Industry standards. After that, the fixed oil was extracted by the first cold pressure from the dried seeds using a mechanical oil press (SMIR, MUV1 65). The seeds were dropped into a cylinder that contains a rotating screw. This screw grinds and crushes the seeds until the oil is extracted. Small holes in the bottom of the cylinder allow the oil to escape into a collection container. The extracted oils were kept in hermetically sealed opaque glass bottles until their use.

“Inflocine®”: topical ointment was purchased from the local pharmacy. It is a nonsteroidal anti-inflammatory indicated for acute inflammation treatment. The remaining chemicals used were of analytical grade.

### 2.2. Animals

The assays of this study were conducted on adult male Wistar rats weighing 168 ± 23 g. The animals were obtained from the local Central Pharmacy, Tunisia.

The experimental protocols were conducted following the guide for the care and use of laboratory animals issued by the University of Sfax, Tunisia, and approved by the Committee of Animal Ethics. The animals were kept in separate cages to avoid licking or biting of inflammatory areas by other animals.

### 2.3. Experimental Protocol

Before inducing the paw edema, a total of 36 rats were divided randomly into six equal groups:

*Group 1:* the rats were normal, and they did not undergo any inflammatory reaction, used as a control group.

*Group 2:* the rats were injected with carrageenan and treated with a normal saline (normal saline-treated group).

*Group 3:* the rats were injected with carrageenan topically treated with a reference drug “Inflocine®” (2 mg/paw) (reference group).

*Groups 4, 5, and 6:* the rats were injected with carrageenan and topically treated with pumpkin, linseed, and prickly pear extracted oils (25 *μ*l/paw), respectively (oil-treated groups). The dose of “25 *μ*l/paw” was experimentally optimized to cover the total paw edema and to grant an anti-inflammatory efficiency.

### 2.4. Model of Carrageenan-Induced Paw Edema

The anti-inflammatory activity was assessed by the *in vivo* experimental model for screening the acute inflammation using carrageenan-induced rat paw [17].

Acute inflammation was induced by the subplantar administration of 100 *μ*l of 1% freshly prepared solution of carrageenan in a normal saline in the right hind paw of each rat.

The paw thickness was measured with a digital caliper at 1, 2, 3, 4, and 5 hours after carrageenan injection. To standardize measurements, they were performed in a transverse technique in the midplantar surface of the rat paw for all time points.

The increase in paw edema at the time (*t*) was calculated using the following formula:

Paw thickness (*t*) − Paw thickness (*t*0), where the paw volume (*t*0) was measured on time 0 before the carrageenan injection.

### 2.5. Blood Sample Collection

Five hours after carrageen induction, the rats were anesthetized by 50 mg/kg ketamine intramuscularly injected, and blood samples were collected with heparin through cardiac puncture. Plasma samples were drawn from blood after centrifugation at 3000 rpm for 15 minutes and were stored at -80°C until biochemical analysis. To eliminate interassay variance, all samples were analyzed in the same assay run. All assays were performed in triplicate in the same laboratory with simultaneous use of a control.

### 2.6. Inflammatory Parameters

#### 2.6.1. Testing the Blood Count

The Sysmex KX-21N analyzer was used to determine the following hematological parameters: white blood cells and platelets.

#### 2.6.2. Inflammatory Biomarkers

*(1) Determination of Protein Markers of Inflammation*. C-reactive protein (CRP) is a specific and reliable biomarker that followed the inflammatory process. It increases in proportion to its intensity [18]. The reactive protein was measured by a turbidimetric method using an automatic analyzer Architect Ci 4100 «ABOTT.» The CRP is expressed with mg/l.

*(2) Fibrinogen Assay of Plasma*. The plasma fibrinogen concentration was determined by the Clauss clotting method [19] measured on the STA® analyzer. The principle test measures the conversion rate of fibrinogen into fibrin in the diluted sample in the presence of an excess of thrombin and records the clotting time. The clotting time is inversely proportional to the level of fibrinogen in the plasma. The fibrinogen level is expressed with g/l plasma.

### 2.7. Oxidative Stress Parameters

#### 2.7.1. Preparation of Cytosolic Extracts

The oxidative stress parameters were determined in skin homogenates diluted in phosphate buffer (pH 7.4) and centrifuged at 9000 rpm for 15 minutes. The resulting supernatants were used for biochemical assays.

#### 2.7.2. Protein Quantification

Skin protein contents were measured according to Bradford method [20] using bovine serum albumin as standard.

#### 2.7.3. Thiobarbituric Acid Reactive Substances (TBARS) Assay

The assay of thiobarbituric acid reactive substances (TBARS) measures malondialdehyde (MDA) present in the sample, as well as malondialdehyde generated from lipid hydroperoxides by the hydrolytic conditions of the reaction. Tissue malondialdehyde concentrations were monitored spectrophotometrically according to the method of Esterbauer [21]. In brief, an aliquot of tissue extract supernatant was mixed with 50 *μ*l of TBS buffer and 125 *μ*l of 20% trichloroacetic acid and centrifuged at 1000 rpm for 10 minutes. A volume of 160 *μ*l of thiobarbituric acid reagent was added to 200 *μ*l of supernatant and 40 *μ*l Tris-HCl (0.6 mol/l) and heated at 80°C for 10 minutes. The mixture was then cooled and measured for absorbance at 532 nm using a spectrophotometer. The malondialdehyde values were calculated using 1,1,3,3-tetraethoxypropane as standard and expressed as nmol of malondialdehyde per gram of protein.

#### 2.7.4. Advanced Oxidation of Protein Products (AOPP) Assay

Advanced oxidation protein product (AOPP) levels were determined according to the method described by Kayali et al. [22]. Briefly, 0.4 ml of the supernatant of tissue homogenate was treated with 0.8 ml phosphate buffer (0.1 mol/l; pH 7.4). After 2 minutes, 0.1 ml of 1.16 mol/l potassium iodide was added to the tube followed by 0.2 ml of acetic acid. The absorbance of the reaction mixture was immediately recorded at 340 nm. The concentration of AOPP is expressed as *μ*moles per milligram of protein.

### 2.8. Antioxidant Enzyme Activities

The activity of antioxidants, namely, superoxide dismutase, catalase, and glutathione peroxidase, was measured in untreated and treated skin tissue homogenate.

#### 2.8.1. Superoxide Dismutase (SOD) Activity Assay

The superoxide dismutase (SOD) activity was estimated according to the method described by Asada et al. [23]. Superoxide radicals are generated in riboflavin, methionine, and illuminate and assayed by the reduction of NBT to form blue formazan (NBT2+). All the solutions were prepared in phosphate buffer (pH 7.4). The photo-induced reactions were performed using fluorescent lamps (20 W). The absorbance was measured at 580 nm. One unit of SOD activity is defined as the amount of enzyme required to cause 50% inhibition of the reduction of NBT measured at 580 nm.

#### 2.8.2. Catalase Activity Assay

The catalase activity was assessed following the method of Aebi [24]. Briefly, 20 *μ*l of homogenate tissue was added to 980 *μ*l of H2O2 solution (containing 0.5 mol/l H2O2 and 0.1 mol/l phosphate buffer). Catalase activity was determined by monitoring the H2O2 decomposition which was measured spectrophotometrically by the decrease in absorbance at 240 nm. Enzyme activity was calculated using a molar extinction coefficient and expressed as *μ*mol H2O2 consumed/min/mg of protein (*μ*M/minute/mg protein).

#### 2.8.3. Glutathione Peroxidase (GPx) Activity Assay

The glutathione peroxidase (GPx) activity was determined according to Flohe and Gunzler method [25]. For the enzyme reaction, 200 *μ*l of tissue homogenates was placed into a tube and mixed with 400 *μ*l glutathione (GSH) and 200 *μ*l of potassium sodium phosphate buffer. After 5 minutes of incubation at 25°C, 200 *μ*l of H2O2 was added, and after 10 minutes, the reaction was terminated by the addition of 1 ml TCA (5%). Then, the mixture was centrifuged at 3000 rpm for 10 minutes, and the supernatant was collected. 480 *μ*l of the supernatant was placed into a cuvette, and 2.2 ml of disodium hydrogen phosphate (Na2HPO4) and 320 *μ*l of 0.4 mg/ml 5,5′-dithio-bis (2-nitrobenzoic acid) (DTNB) were added for color development. The absorbance at wavelength 412 nm was measured. The enzyme activity was calculated as a decrease in GSH within the reaction time as compared to that in the nonenzyme reaction and expressed as *μ*moles of GSH/min/mg of protein.

### 2.9. Histological Evaluation

Five hours after carrageenan injection, the rats were sacrificed and autopsy samples were taken from the middle of the treated paw tissues for histopathological assessment. The sample tissues were fixed in neutral buffered formalin solution (10%), embedded in paraffin wax, cut into 5-*μ*m-thick sections, and stained with hematoxylin-eosin. The slides were photographed with an Olympus U-TU1X-2 camera connected to an Olympus CX41 microscope (Tokyo, Japan).

### 2.10. Statistical Analysis

The data obtained were analyzed via SPSS software. Quantitative variables were presented as the mean ± standard deviation (SD) for each group. Statistical comparisons between the groups were carried out using ANOVA followed by the Tukey test. Differences between groups were considered statistically significant at *p* value < 0.05.

## 3. Results

### 3.1. Size of Paw Edema

**Figure 1:**
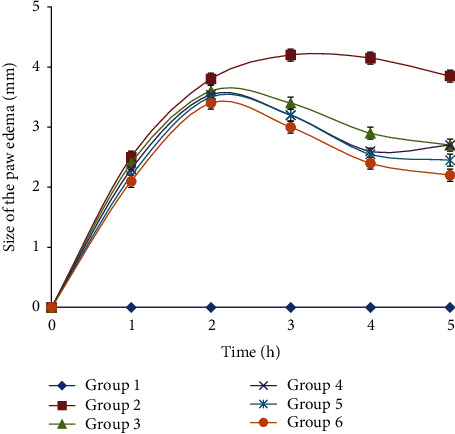
The evolution size of the paw edema during 5 hours after carrageenan injection in different experimental rat groups. All values are mean ± S.D. (*n* = 6/group). Group 1: normal rats (control group); group 2: normal saline-treated group; group 3: “Inflocine®”-treated rats (reference group); group 4: pumpkin oil-treated group; group 5: linseed oil-treated group; group 6: prickly pear oil-treated group.

The evolution size of the edema of each studied group was monitored during 5 hours after carrageenan injection. The results are shown in Figure 1.

The injection of the carrageenan at the front part of the right paw led to an increase in the paw thickness. The maximum size was observed 3 hours after carrageenan injection. A significant reduction in the size of the edema was observed in the oil-treated group. This reduction was better than that of the rats of the reference group. Prickly pear oil-treated group resulted in a significant increase in the paw edema size compared to all the studied groups.

### 3.2. White Blood Cells and Platelets Numbers

**Figure 2:**
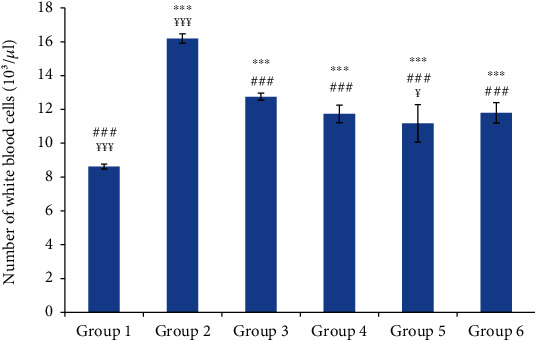
The number of white blood cells in different experimental rat groups five hours after carrageenan injection. All values are mean ± S.D. (*n* = 6/group). Group 1: normal rats (control group); group 2: normal saline-treated group; group 3: “Inflocine®”-treated rats (reference group); group 4: pumpkin oil-treated group; group 5: linseed oil-treated group; group 6: prickly pear oil-treated group. ^∗∗∗^*p* ≤ 0.001: highly significant difference compared to group 1; ^###^*p* ≤ 0.001: highly significant difference compared to group 2; ^¥¥¥^*p* ≤ 0.001: highly significant difference compared to group 3; ^¥^*p* ≤ 0.05: significant difference compared to group 3.

**Figure 3:**
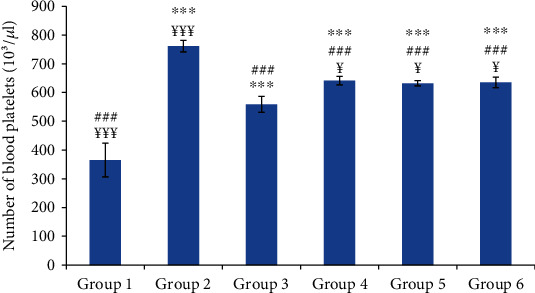
The number of blood platelets in different experimental rat groups five hours after carrageenan injection. All values are mean ± S.D. (*n* = 6/group). Group 1: normal rats (control group); group 2: normal saline-treated group; group 3: “Inflocine®”-treated rats (reference group); group 4: pumpkin oil-treated group; group 5: linseed oil-treated group; group 6: prickly pear oil-treated group. ^∗∗∗^*p* ≤ 0.001: highly significant difference compared to group 1; ^###^*p* ≤ 0.001: highly significant difference compared to group 2; ^¥¥¥^*p* ≤ 0.001: highly significant difference compared to group 3; ^¥^*p* ≤ 0.05: significant difference compared to group 3.

Hematological parameters were assessed by monitoring the numbers of white blood cells and blood platelets. As shown in Figures 2 and 3, the numbers of white blood cells and platelets were significantly increased in group 2 (normal saline-treated group) compared to the control group. In comparison with group 2, the oil-treated groups and the reference group showed a significant decrease in white blood cell (WBC) and platelet counts without reaching the normal counts of the control group.

### 3.3. Inflammatory Biomarkers: CRP and Fibrinogen Concentrations

**Figure 4:**
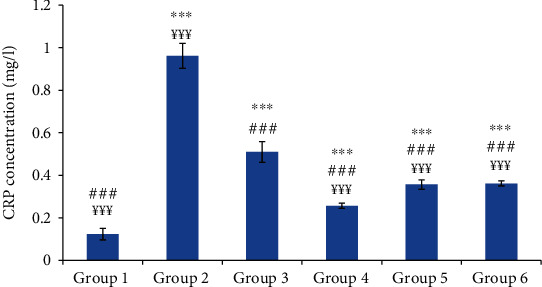
C-reactive protein (CRP) concentration in different experimental rat groups five hours after carrageenan injection. All values are mean ± S.D. (*n* = 6/group). Group 1: normal rats (control group); group 2: normal saline-treated group; group 3: “Inflocine®”-treated rats (reference group); group 4: pumpkin oil-treated group; group 5: linseed oil-treated group; group 6: prickly pear oil-treated group. ^∗∗∗^*p* ≤ 0.001: highly significant difference compared to group 1; ^###^*p* ≤ 0.001: highly significant difference compared to group 2; ^¥¥¥^*p* ≤ 0.001: highly significant difference compared to group 3.

**Figure 5:**
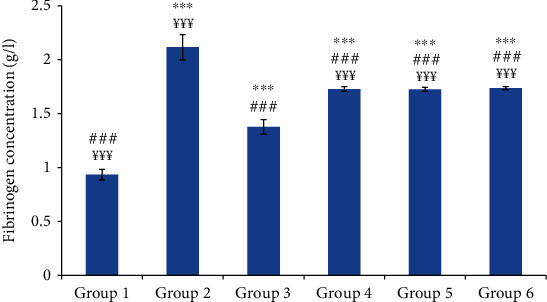
The fibrinogen concentration in different experimental rat groups five hours after carrageenan injection. All values are mean ± S.D. (*n* = 6/group). Group 1: normal rats (control group); group 2: normal saline-treated group; group 3: “Inflocine®”-treated rats (reference group); group 4: pumpkin oil-treated group; group 5: linseed oil-treated group; group 6: prickly pear oil-treated group. ^∗∗∗^*p* ≤ 0.001: highly significant difference compared to group 1; ^###^*p* ≤ 0.001: highly significant difference compared to group 2; ^¥¥¥^*p* ≤ 0.001: highly significant difference compared to group 3.

Compared to the control group, group 2 (normal saline-treated group) showed a highly significant increase in mean blood concentrations of CRP and fibrinogen. The oil-treated groups and the reference group showed a significant decrease in the concentration of these inflammatory biomarkers compared to those of group 2; however, these concentrations did not reach the concentration of the control group (Figures 4 and 5).

### 3.4. Assessment of Lipid Peroxidation

**Figure 6:**
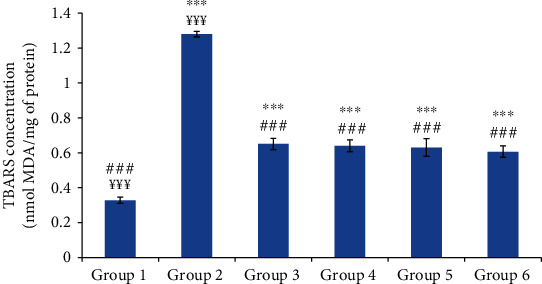
The skin thiobarbituric acid reactive substances (TBARS) concentration of different experimental rat groups five hours after carrageenan injection. All values are mean ± S.D. (*n* = 6/group). Group 1: normal rats (control group); group 2: normal saline-treated group; group 3: “Inflocine®”-treated rats (reference group); group 4: pumpkin oil-treated group; group 5: linseed oil-treated group; group 6: prickly pear oil-treated group. ^∗∗∗^*p* ≤ 0.001: highly significant difference compared to group 1; ^###^*p* ≤ 0.001: highly significant difference compared to group 2; ^¥¥¥^*p* ≤ 0.001: highly significant difference compared to group 3.

The results of the skin lipid peroxidation are monitored in Figure 6. Data showed that carrageenan injection led to a significant increase in skin TBARS concentration. Compared to group 2, the oil-treated groups and the reference group exhibited a significant decrease (*p* ≤ 0.001) in skin TBARS concentrations. The TBARS concentrations of the oil-treated groups were similar to that of the reference group without reaching the normal concentration.

### 3.5. Assessment of Advanced Oxidized Protein Products (AOPP)

**Figure 7:**
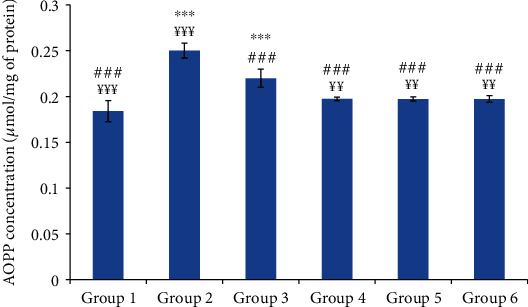
The skin advanced oxidation protein product (AOPP) concentration of different experimental rat groups. All values are mean ± S.D. (*n* = 6/group). Group 1: normal rats (control group); group 2: normal saline-treated group; group 3: “Inflocine®”-treated rats (reference group); group 4: pumpkin oil-treated group; group 5: linseed oil-treated group; group 6: prickly pear oil-treated group. ^∗∗∗^*p* ≤ 0.001: highly significant difference compared to group 1; ^###^*p* ≤ 0.001: highly significant difference compared to group 2; ^¥¥¥^*p* ≤ 0.001: highly significant difference compared to group 3; ^¥¥^*p* ≤ 0.05: very significant difference compared to group 3.

After acute inflammation, proteins are susceptible to oxidative modifications that lead to the production of carbonylated proteins. The skin AOPP concentrations are illustrated in Figure 7. The concentration of the advanced oxidized protein products was significantly higher in group 2 (normal saline-treated group) than in the control group. The topical application of the tested oils seemed to significantly reduce the skin AOPP concentration.

### 3.6. Exploring the Antioxidant Status

#### 3.6.1. Superoxide Dismutase (SOD) Activity

**Figure 8:**
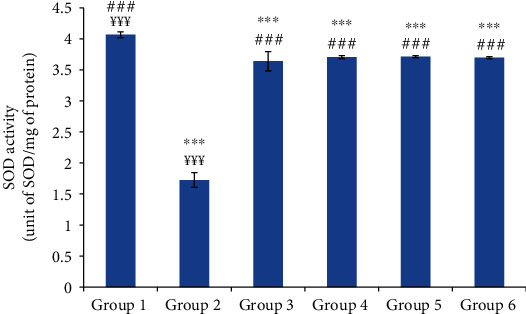
The skin superoxide dismutase (SOD) activity of different experimental rat groups. All values are mean ± S.D. (*n* = 6/group). Group 1: normal rats (control group); group 2: normal saline-treated group; group 3: “Inflocine®”-treated rats (reference group); group 4: pumpkin oil-treated group; group 5: linseed oil-treated group; group 6: prickly pear oil-treated group. ^∗∗∗^*p* ≤ 0.001: highly significant difference compared to group 1; ^###^*p* ≤ 0.001: highly significant difference compared to group 2; ^¥¥¥^*p* ≤ 0.001: highly significant difference compared to group 3.

The injection of the carrageenan led to a significant decrease in skin SOD activity of 57.58% in group 2 (normal saline-treated group) compared to the control group (*p* ≤ 0.001). However, the oil-treated groups showed a significant increase in skin SOD activity compared to group 2. There was no significant difference between the skin SOD activities of the oil-treated groups and the reference group (Figure 8).

#### 3.6.2. Catalase (CAT) Activity

**Figure 9:**
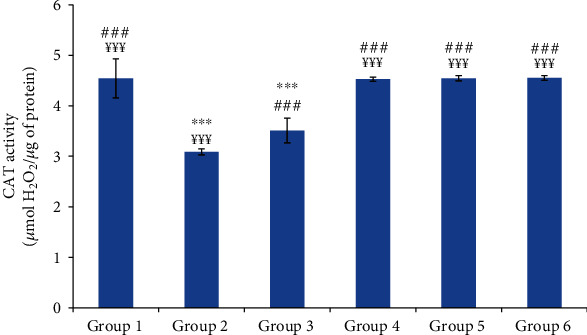
The skin catalase (CAT) activity of different experimental rat groups. All values are mean ± S.D. (*n* = 6/group). Group 1: normal rats (control group); group 2: normal saline-treated group; group 3: “Inflocine®”-treated rats (reference group); group 4: pumpkin oil-treated group; group 5: linseed oil-treated group; group 6: prickly pear oil-treated group. ^∗∗∗^*p* ≤ 0.001: highly significant difference compared to group 1; ^###^*p* ≤ 0.001: highly significant difference compared to group 2; ^¥¥¥^*p* ≤ 0.001: highly significant difference compared to group 3.

Group 2 (normal saline-treated group) revealed a significant decrease in the skin catalase activity of 32.53% compared to the control group. However, the skin catalase activities of the oil-treated groups were similar to that of control and reference groups (Figure 9).

#### 3.6.3. Glutathione Peroxidase (GPx) Activity

**Figure 10:**
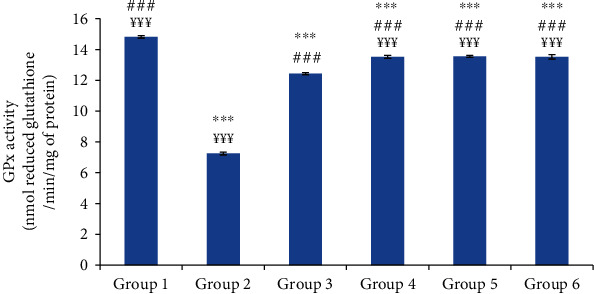
The skin glutathione peroxidase (GPx) activity of different experimental rat groups. All values are mean ± S.D. (*n* = 6/group). Group 1: normal rats (control group); group 2: normal saline-treated group; group 3: “Inflocine®”-treated rats (reference group); group 4: pumpkin oil-treated group; group 5: linseed oil-treated group; group 6: prickly pear oil-treated group. ^∗∗∗^*p* ≤ 0.001: highly significant difference compared to group 1; ^###^*p* ≤ 0.001: highly significant difference compared to group 2; ^¥¥¥^*p* ≤ 0.001: highly significant difference compared to group 3.

The injection of the carrageenan induced a significant decrease in the skin GPx activity of 51.07% compared to the control group. However, the oil-treated groups depicted a significant increase in the skin GPx activity compared to group 2. The skin GPx activities of the oil-treated groups were similar to that of the reference group (Figure 10).

### 3.7. Histopathological Examination

**Figure 11:**
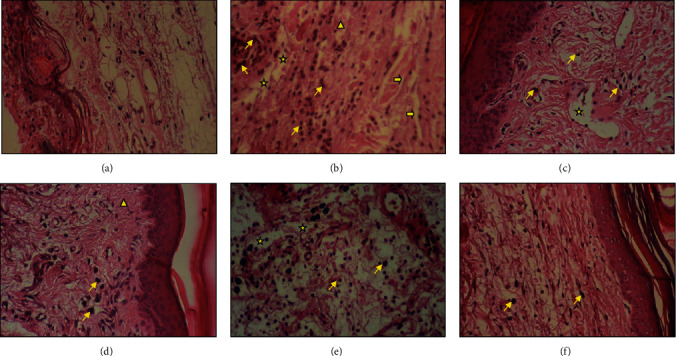
The histological features of the skin sections (×400) of each group stained with hematoxylin-eosin five hours after carrageenan induction. (yellow star): edema; (yellow arrow): dilated vessel; (yellow slant arrow): inflammatory nucleus; (yellow triangle): vacuum cells. (a) Histological section of a normal skin area of representative control rat (control group); (b) histological section of representative normal saline-treated rat (group 2); (c) histological section of representative “Inflocine®”-treated rat (group3); (d) histological section of representative pumpkin oil-treated rat (group 4); (e) histological section of representative linseed oil-treated rat (group 5); (f) histological section of representative prickly pear oil-treated rat (group 6).

The microscopic examination of the skin specimen of group 2 (normal saline-treated group) revealed the presence of interstitial edema; the predominance of inflammatory cells in the deep dermis associated with vascular congestion (hyperemia) and dilated capillaries. However, the skin specimen of the oil-treated groups and the reference group showed a significant decrease in inflammatory cells in the dermis and inside the blood vessels. These inflammatory signs seemed to be less significant in prickly pear oil-treated biopsies (Figure 11).

## 4. Discussion

The carrageenan-induced paw edema in rat is an acute inflammation model widely used for the screening of synthetic or natural anti-inflammatory products [26]. The acute inflammatory response is a protective mechanism of the body to remove the injurious stimuli that involve local cellular and vascular processes following tissue damage [1].

In this respect, this study aims at assessing the anti-inflammatory effect of pumpkin, linseed, and prickly pear extracted oils using the carrageenan-induced paw model compared to a nonsteroidal anti-inflammatory drug “Inflocine®.” As oxidative stress plays a key role in inflammatory processes, the tested oils were assessed for their effect on inflammatory mediators and oxidative stress markers in acute inflammation.

In comparison to the control group, we noticed that the normal saline-treated group had a significant paw thickness increase which peaked at 3 hours after carrageenan injection. This finding was also described by Umamageswari and Maniyar [27]. Indeed, carrageenan induced an acute inflammatory reaction typically characterized by vasodilation and tissue swelling. This inflammatory exudate is mainly composed of polymorphonuclear leukocytes [28]. The acute inflammatory response is actually triggered by the release of mediators such as histamine and serotonin in the first few hours. The latter are related to the release of prostaglandins, proteases, and superoxide radicals. These mediators are responsible for the vasodilation of the vessels, the vascular hyperpermeability, the redness, and the edema [29, 30].

Three hours after carrageenan injection, the reference group and the oil-treated groups showed a significant reduction in the paw thickness compared to the carrageenan treated group. Indeed, it was reported that the injection of carrageenan induced a peak in COX-2 concentration one hour after the injection. This peak was associated with an increase in the synthesis of prostaglandins involved in the inflammatory process [31]. This may explain the late effect of nonsteroidal anti-inflammatory drugs such as “Inflocine®” whose effect appeared only after 2 hours. This effect was related to its mode of action which involves the inhibition of prostaglandin synthesis induced by COX-1 and COX-2 [32].

The inflammatory reaction is coordinated by various mediators that involve cell recruitment to the site of inflammation. This leads to investigating the inflammatory parameters (including CRP and fibrinogen), white blood cells, and blood platelets.

The plasma concentrations of fibrinogen and CRP were significantly increased in the normal saline-treated group compared to the control group. The CRP and fibrinogen are the protein biomarkers of inflammation that are increased during the acute inflammation [33]. Indeed, the CRP which has proinflammatory and prothrombotic effects increases the expression of tissue factor and interleukin-6. The acute inflammation was also associated with an increase of white blood cell (WBC) and platelet counts. This leukocytosis was attributed to the increase of leukocyte recruitment and could be directly proportional to the severity of the causative stress condition [34]. It was reported that carrageenan stimulated immune functions by the infiltration of WBC into the site of inflammation due to the release of proinflammatory cytokines (IL-I). The latter increased the production of both granulocyte and macrophages colony-stimulating factors [35].

The significant decrease of the hematological parameters and the clinical signs in the oil-treated groups as well as in the reference group compared to the control group suggest a potent anti-inflammatory effect of the tested oils. It was reported that the anti-inflammatory drugs depleted the migration of inflammatory cells by inhibiting the release of various chemical mediators [36].

The carrageenan injection not only induced significant increase in TBARS concentration and the AOPP concentrations but also resulted in a significant decrease of the activities of antioxidant enzymes (SOD, CAT, GPx). Indeed, the phospholipid bilayer of the cell membrane, which is particularly rich of polyunsaturated fatty acids and proteins, formed a target for free radical attack resulting in an increase in skin TBARS and AOPP concentrations [37]. The decrease in the activities of the antioxidant enzymes such as SOD, CAT, and GPx might be due to their excessive secondary consumption of free radicals which were generated by inflammation. Previous researches revealed that carrageenan injection caused excessive production of free radical. The involvement of oxidative stress in the acute inflammatory process had been already described by several authors [1, 38–40]. The tested oils', once in contact with the skin, lipophilic compounds (tocopherols, sterols, and polyunsaturated fatty acids) seeped into the stratum corneum by percutaneous intercellular absorption and then joined the epidermis and the dermis [41, 42]. The latter was rich in dilated blood vessels which allowed the absorption of bioactive compounds from the oil [43]. The anti-inflammatory effect of the tested oils based on the clinical and biological assessments could be related to their unsaturated fatty acids which were considered as highly active anti-inflammatory compounds acting as inhibitors of cyclo-oxygenases [44, 45]. This effect could be also attributed to the antioxidant properties of the tested oils for their bioactive compounds such as phytosterols, tocopherols, polyphenols, and carotenoids. In fact, the lipophilic aspect of these compounds allowed them to diffuse inside the cell membrane which is rich in polyunsaturated fatty acids and protein. Then, these bioactive compounds might trap the activated oxygen species; as a consequence, the spread of both lipid and protein oxidations might be inhibited. Added to that, it was described that phytosterols, in particular *β*-sitosterols, and polyphenols had also anti-inflammatory activities [46].

The prickly pear oil provided a better anti-inflammatory effect compared to all the tested oil. This result was closely correlated with its chemical profile, which exhibited a richness in the tocopherols and sterols [45].

## 5. Conclusions

Overall, the data of the current study highlight the anti-inflammatory effect of pumpkin, linseed, and especially prickly pear extracted oils on acute inflammation through the decrease of clinical signs of inflammation, infiltration of inflammatory cells, vascular congestion, and biochemical markers. These anti-inflammatory effects elicited by the tested oils are closely associated with the amelioration of the endogenous skin antioxidant status. Thus, the tested oils may be potentially used as an anti-inflammatory component of new drugs for the treatment of acute inflammation without side effects. However, further studies are necessary to determine the cytokine profile and the lipid mediators of the oil-treated groups.

## Data Availability

No data were used to support this study.
